# Clinical Effects of Thai Herbal Compress: A Systematic Review and Meta-Analysis

**DOI:** 10.1155/2015/942378

**Published:** 2015-03-12

**Authors:** Teerapon Dhippayom, Chuenjid Kongkaew, Nathorn Chaiyakunapruk, Piyameth Dilokthornsakul, Rosarin Sruamsiri, Surasak Saokaew, Anchalee Chuthaputti

**Affiliations:** ^1^Department of Pharmacy Practice, Faculty of Pharmaceutical Sciences, Naresuan University, Phitsanulok 47500, Thailand; ^2^Center of Pharmaceutical Outcomes Research, Department of Pharmacy Practice, Faculty of Pharmaceutical Sciences, Naresuan University, Phitsanulok 65000, Thailand; ^3^Center of Excellence for Environmental Health and Toxicology, Naresuan University, Phitsanulok, Thailand; ^4^School of Pharmacy, Monash University Malaysia, Kuala Lumpur, Malaysia; ^5^School of Population Health, University of Queensland, Brisbane, QLD 4006, Australia; ^6^School of Pharmacy, University of Wisconsin-Madison, Madison, WI 53705, USA; ^7^Center of Health Outcomes Research and Therapeutic Safety (Cohorts), School of Pharmaceutical Sciences, University of Phayao, Phayao 56000, Thailand; ^8^Department for Development of Thai Traditional and Alternative Medicine, Ministry of Public Health, Nonthaburi 11000, Thailand

## Abstract

*Objective*. To determine the clinical effects of Thai herbal compress. *Methods*. International and Thai databases were searched from inception through September 2014. Comparative clinical studies investigating herbal compress for any indications were included. Outcomes of interest included level of pain, difficulties in performing activities, and time from delivery to milk secretion. Mean changes of the outcomes from baseline were compared between herbal compress and comparators by calculating mean difference. *Results*. A total of 13 studies which involved 778 patients were selected from 369 articles identified. The overall effects of Thai herbal compress on reducing osteoarthritis (OA) and muscle pain were not different from those of nonsteroidal anti-inflammatory drugs, knee exercise, and hot compress. However, the reduction of OA pain in the herbal compress group tended to be higher than that of any comparators (weighted mean difference 0.419; 95% CI −0.004, 0.842) with moderate heterogeneity (*I* ^2^ = 58.3%, *P* = 0.048). When compared with usual care, herbal compress provided significantly less time from delivery to milk secretion in postpartum mothers (mean difference −394.425 minutes; 95% CI −620.084, −168.766). *Conclusion*. Thai herbal compress may be considered as an alternative for osteoarthritis and muscle pain and could also be used as a treatment of choice to induce lactation.

## 1. Introduction

Herbal compresses or “Luk Prakob” in Thai have been used in Thailand for hundreds of years in conjunction with traditional massage or as a stand-alone therapy [1]. It was approved and listed in the National List of Essential Medicines for the relief of muscle sprains and muscular and joint pain [2]. Herbal compress service is now provided for therapeutic and rehabilitative purposes in most public health service facilities in Thailand and is a Thai traditional medicine treatment modality covered by the country's health security systems. In addition, hot herbal compress is also commonly found in spa industry in Thailand and abroad as a part of the spa and massage services [3]. It is suggested that the composite effects of herbal compress derived from (1) heat conduction to increase regional blood flow to the affected areas, (2) anti-inflammatory effects of herbal ingredients, and (3) relaxation effects of aromatic volatile oil [4]. To achieve these effects, herbal compresses must be steamed for 10–15 minutes before their use to enable heat conduction and to facilitate the release of active substances and volatile oils from herbal ingredients.

The ingredients of Thai herbal compress vary widely and depend on the availability of herbs in different local areas and the unique formula of individual recipe [5]. Generally, the main herbal ingredients of most herbal compresses include Plai or Cassumunar ginger (*Zingiber cassumunar* Roxb. or* Zingiber montanum* (J. Koenig) Link ex A. Dietr.), turmeric (*Curcuma longa* L.), and camphor. These herbal components have long been used solely or partly for the treatment of several inflammatory conditions in many traditional medicines including Ayurveda which is almost six thousand years old [6]. Findings from several published literature have also endorsed the anti-inflammatory and analgesic effects of* Zingiber montanum* [7],* Curcuma longa* [8], and camphor [9].

Thai herbal compress has been selected in 2013 as one of the five champion herbal products that have been widely used and generated income to the country [23]. A number of research studies have been conducted to evaluate the effects of herbal compress in a variety of clinical conditions such as osteoarthritis (OA) [10], myofascial pain [16], labor pain [12], and insufficient/delayed lactation [20]. However, there is a gap in the literature with no summarization of evidence on the clinical benefits of Thai herbal compress. Although a recent Cochrane systematic review has gathered evidence on topical herbal therapies for treating osteoarthritis, which included* Arnica* gel,* Capsicum* extract gel, comfrey extract gel, Chinese herbal patches, Fufang Nanxing Zhitong Gao (FNZG) patches, Shangshi Jietong Gao (SJG) patches, Marhame-Mafasel compress, and stinging nettle leaf, the use of Thai herbal compress was not covered in this review [24]. The objective of this study is therefore to systematically review and determine the clinical effects of Thai herbal compress in all identified indications.

## 2. Methods

This systematic review was conducted according to the Cochrane Collaboration framework guidelines [25], and the reporting follows the PRISMA Statement [26].

### 2.1. Search Strategies and Study Selection

The following databases were used to search for original research articles from inception to September 2014: AMED, CINAHL, Cochrane Central Register of clinical trial, EMBASE, Health Science Journals in Thailand, PubMed, Thai Index Medicus, Thai Library Integrated System, Thai Medical Index, Thai Thesis Database, WHO registry, and https://www.clinicaltrial.gov/. Strategic search terms used were Herbal compress^*^ OR Herbal ball OR Herbal dabber OR “Luk PraKob” (a Thai word for herbal compress). References of papers derived for full text review were scanned to identify potential studies not indexed in the above databases.

Research articles were included if they met the following inclusion criteria: (1) conducted in human; (2) evaluated clinical effects of Thai herbal compress; and (3) had control group. TD scanned all the titles and abstracts to determine whether the studies assessed the effects of herbal compress. Full-text articles of the potential studies were subsequently assessed by TD and CK. When disagreements and uncertainties regarding eligibility occurred, they were resolved by discussions with NC.

### 2.2. Data Extraction and Quality Assessment

Data extraction was undertaken by TD and CK using a data extraction form in accordance with the CONSORT statement for reporting herbal medicinal interventions [27]. The data extracted and reported included study design; number of participants; age of participants; herbal compress ingredients; characteristics of the intervention; and outcome measurement. Outcomes of interest depended on indication of herbal compress. For example, outcome measures for studies on pain reduction were level of pain and difficulties in performing activities. Time to milk secretion or milk ejection score was outcomes of interest for studies that investigated the effects of herbal compress on the induction of lactation. Studies included in this review were assessed for methodological quality by TD and CK using the Cochrane risk of bias tool [25] and Jadad score [28]. The Cochrane risk of bias evaluates bias in intervention studies based on a number of criteria including sequence generation; allocation concealment; blinding; incomplete outcome data; selective reporting; and other sources of bias. Studies in which baseline characteristics were different among study groups or not tested for their differences were considered as high risk for the domain of “other risks of bias.” The overall risk of bias for each study was based on the risk of bias of key domains which, in this review, were “sequence generation” and “other sources of bias”. Each study was classified as having low risk (low risk of bias for all key domains), high risk (high risk of bias for one or more key domains), or unclear risk (unclear risk of bias for one or more key domains). Disagreements between the reviewers were settled through discussion and consensus.

### 2.3. Statistical Analysis

Data from all studies were pooled in a meta-analysis to determine the overall effect size with 95% confidence interval. Studies that employed other procedures/medications in addition to herbal compress in the intervention arm or used different measures were excluded from meta-analyses. Pooled effects were calculated and stratified according to indications of herbal compress and its comparators. In addition to the pooled effects of individual comparators (such as NSAIDs, knee exercise, and hot compress) a collection of all comparators was formulated for each treatment indication and called a “combined comparator.” The combined comparator analyses were undertaken to examine the overall impact of herbal compress for each outcome regardless of comparative treatments. To avoid duplication of studies with more than one comparator arms, only one comparator was selected from each study to be included in a combined comparators analysis. The following are order of preferences used to choose the best comparator for each indication: (a) OA: NSAIDs (nonsteroidal anti-inflammatory drugs), knee exercise, and hot compress [29] and (b) myofascial pain: NSAIDs and hot compress [30].

Mean changes of the outcome variables for each treatment arm were calculated by subtracting the baseline mean with the mean of the postintervention. Pooled standard deviations (*S*_pooled_) of the mean changes were used [31]. Then mean changes of the outcome variables were compared between intervention and comparator arms by calculating the overall mean differences, which could be (1) standardized mean difference (SMD) for outcomes that were measured by different scales across studies or (2) weighted mean difference (WMD) for outcomes that were measured on the same scale. For pain reduction indication, the overall mean difference of outcome above 0 indicated that herbal compress was more effective in reducing pain or alleviating difficulties in performing activities compared to comparators. For the use of herbal compress to promote milk secretion, an overall mean difference value lower than 0 suggested that the duration from delivery to lactation in herbal compress arm was lower than that in the comparator arm which signify its beneficial effects.

Statistical heterogeneity between studies was assessed using the chi-squared test and *I* ^2^. Thresholds of *I* ^2^ were interpreted in accordance with the magnitude and direction of effects and strength of evidence of heterogeneity (e.g., *P* value) as follows: might not be important (0%–40%); moderate heterogeneity (30%–60%); substantial heterogeneity (50%–90%); and considerable heterogeneity (75%–100%) [25]. The Dersimonian and Laird random-effects model [32] was employed for all analyses. Meta-analyses were conducted using STATA version 10 (STATA Corp., College Station, TX, USA).

## 3. Results

### 3.1. Study Selection

**Figure 1:**
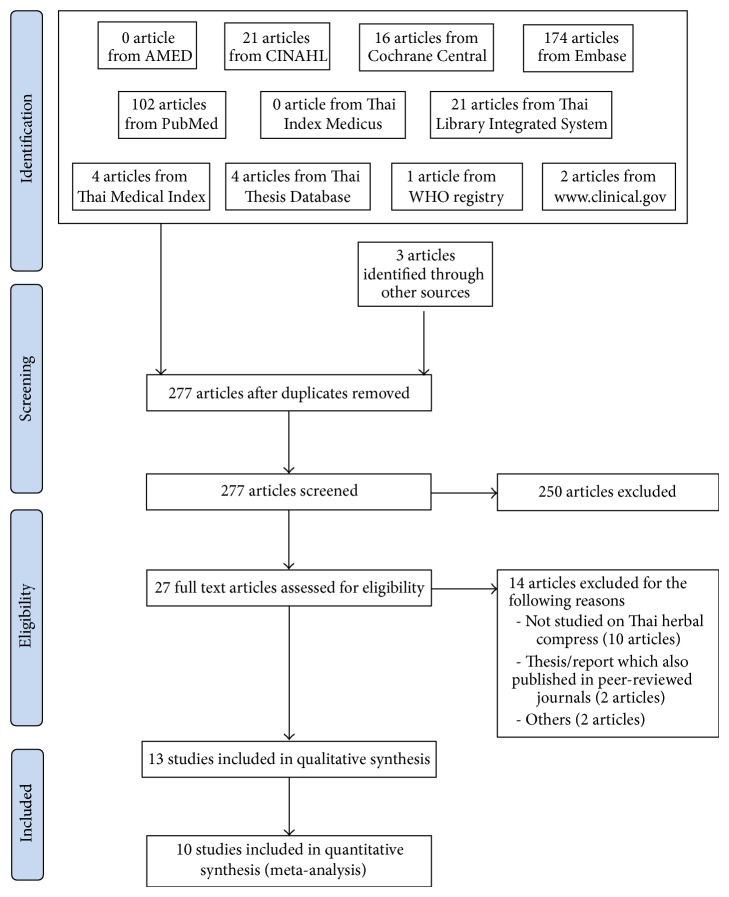
Flow diagram of selected articles.

Of the 363 articles extracted from the various databases searched and 3 articles identified through other sources, 277 articles were eligible for screening after duplication removal. Based on title and abstract screened, 27 articles were selected for full text review. A total of 14 papers were excluded after full text review as 10 studies did not use Thai herbal compress recipe, 2 studies were thesis/report which were also published in peer-reviewed journals, and the remaining 2 studies were not clinical studies and did not study the effect of herbal compress. Eventually, 13 studies [10–22], which involved a total of 778 patients, met the inclusion criteria for this review (Figure 1).

### 3.2. Study Characteristics

The indications of herbal compress used in these studies were pain reduction [10–19] and induction of lactation [20–22]. For studies that investigated the effects of Thai herbal compress on pain reduction, five studies [11, 14, 15, 18, 19] were conducted in patients with knee OA, two studies each were conducted in patients with muscle pain [16, 17] and labor pain [12, 13], and one study [10] was undertaken in patients with knee OA and patients with muscle pain (Table 1). The majority of the included studies, 11 out of 13, used a quasi-experiment approach [10, 12–15, 17–22]. The remaining studies were randomized controlled trial [11] and crossover trial [16]. All 13 studies were conducted in Thailand.

The ingredients of Thai herbal compress were reported in 9 studies [10, 11, 13–17, 19, 22]. Although a variation in the proportion of herbal ingredients was observed, the main components of herbal compress in these nine studies appeared similar and included* Zingiber montanum*,* Curcuma longa*, and camphor. The duration for steaming the herbal compress before application ranged from 10 to 20 minutes. The intervention group in 10 studies [11, 13–21] received herbal compress as stand-alone therapy. Other studies used herbal compress in conjunction with massage [12, 22] or massage and NSAIDs [10].

Comparators that were most used among studies in patients with knee OA and muscle pain were hot compress (4 studies) [14, 16, 17, 19], followed by NSAIDs (3 studies) [10, 11, 17]. Knee exercise was used in two studies [14, 18] and oil massage and modern medicine was used in one study [15]. Usual labor care was used as a comparator in both studies in postpartum mothers with labor pain [12, 13]. For studies on the induction of lactations, two studies [20, 21] used routine milk production program which included baby holding and breast feeding promotion every 2-3 hours as a comparator, while breast massage followed by hot compress was used in one study [22].

All three studies investigating the effects of herbal compress on improving lactation assessed the outcome after a single course treatment [20–22]. For those using herbal compress for pain reduction, three studies [12, 13, 16] assessed clinical outcomes after a single course therapy. The remaining studies measured the effects of herbal compress after a number of treatment sessions which ranged from 3 [17] to 14 sessions [18], with the duration spanning from 5 days [10, 14, 17] to 4 weeks [15]. Seven studies [10–12, 16–18, 22] stated that herbal compresses were applied by trained/experiences health personnel or traditional medicine practitioners. Of note is that the investigators were the ones who apply herbal compress to their participants in three studies [13, 19, 20]. The remaining studies [14, 15, 21] did not clearly state the characteristics of practitioners who treated intervention groups with herbal compress.

### 3.3. Quality of Included Studies

The majority of included studies, 10 out of 13 [10, 12, 14, 15, 17–22], were prone to risk of bias as they used quasi-experimental designs that failed to randomize participants to receive herbal compress intervention (Table 2). For those that conducted sequence generation [11, 13, 16], only one study reported allocation concealment [11]. Considering the nature of this intervention, it was not feasible to undertake a blinding method in all studies. Bias in incomplete outcome data was observed in one study [22] and another study [12] showed bias in selective outcome reporting. Eleven studies [10, 13–22] reported difference in baseline characteristics among study groups or were not tested for their differences and therefore were considered as having high risk for the domain of “other sources of bias.” The overall risk of bias within the studies, based on the risk of bias of key domains, yielded only one study with a low risk of bias [11], which was consistently confirmed by its high Jadad score.

Information extracted from the included studies was generally complied with the requirement in the CONSORT statement for reporting herbal medicinal interventions [27], except information related to herbal ingredients. It was found that only one study reported the Latin binomial of ingredient herbs [11]. Method of authentication of raw material was also reported in one study [22], and no studies had undertaken quality testing of herbal ingredients used in herbal compress.

### 3.4. Clinical Effects of Herbal Compress in Patients with Knee OA

Of the studies that investigated the use of herbal compress in patients with knee OA, all six studies [10, 11, 14, 15, 18, 19] measured the level of pain using visual analogue scale (VAS). Five [10, 11, 14, 18, 19] of these studies also evaluated difficulty in performing selected activities (Table 3). Elderly patients aged over 60 years were specified as the subjects of investigation in two studies [15, 18]. The mean age of subjects in the remaining studies was lower than 60 years in two studies [10, 14] and over than 60 years in one study [11], and one study reported the age of subjects ranging from 40 to 79 years old.

Variations in measuring level of pain and reporting scale were observed among these studies. Most studies [10, 14, 15, 18, 19] requested patients to record their current pain level when performing certain activities, whilst one study [11] asked patients to rate their pain over the past two days. The number of activities that patients were asked to assess their pain also varied widely from unspecified [10, 15] to 15 activities [18]. Average VAS of pain with the scale range from 0 to 10 was reported in four studies [10, 14, 15, 18], whereas the other two studies reported total pain with the scale range of 0–130 [19] and 0–500 [11]. Overall, the average baseline OA pain of subjects in two studies was lower than 50% of the maximum VAS score [15, 18], between 50% and 59.9% in three studies [10, 11, 14], and 60% in one study [19].

Two studies [10, 12] used other treatments in conjunction with herbal compress in the intervention group and thus were not included in a meta-analysis. The overall effect sizes of herbal compress to reduce OA pain were not different when compared with NSAIDs, knee exercise, and hot compress, that is, with SMD of 0.35 (95% CI −0.09, 0.79), WMD of 0.67 (95% CI −0.84, 2.16), and SMD of 0.25 (95% CI −0.16, 0.67), respectively (Table 4). However, the reduction of OA pain from baseline in herbal compress group tended to be higher than combined comparators regardless of treatment options used (standardized mean difference 0.419; 95% CI −0.004, 0.842) with moderate heterogeneity across studies (*I* ^2^ = 58.3%, *P* = 0.048).

For those that evaluated difficulties in performing activities in patients with knee OA, four studies [10, 14, 18, 19] used VAS measure and one study [11] used Lequesne's functional index as a measuring tool. No significant differences were identified for the effect of herbal compress in improving difficulty in performing activities when compared with knee exercise (WMD, −0.001; 95% CI −0.842, 0.840), hot compress (SMD, 0.22; 95% CI −0.43, 0.87), and combined comparators (SMD, 0.22; 95% CI −0.13, 0.58).

### 3.5. Clinical Effects of Herbal Compress in Patients with Muscle Pain

The VAS of pain level with the same rating scale, 0 to 10, was used in all studies on muscle pain [10, 16, 17]. The average baseline pain (mean ± SD) varied among these studies from 3.4 ± 2.4 [16] to 6.1 ± 1.3 [10]. The comparators included NSAIDS [10, 17] and hot compress [16, 17]. However, one out of the two studies that compare herbal compress with NSAIDs did not meet the inclusion criteria for meta-analysis since the intervention group received NSAIDs in addition to herbal compress. Hence, the overall effects of herbal compress in improving muscle pain compared with NSAIDs were not available in this review. Results from meta-analysis on other comparators indicated that herbal does not provide beneficial effects over hot compress (0.52; 95% CI −0.84, 1.89) and combined comparators (0.14; 95% CI −0.57, 0.84) in the treatment of muscle pain.

### 3.6. Clinical Effects of Herbal Compress in Patients with Labor Pain

The overall effect of herbal compress on labor pain reduction could not be drawn because the two studies on this indication were not comparable. One study [12] used herbal compress in conjunction with a Thai traditional massage whereas the other [13] used herbal compress as a stand-alone treatment. However, finding from the individual studies indicated that when compared with usual care, which included medication and exercise, herbal compress significantly reduced labor pain in postpartum mothers who had back pain after labor (the difference of the median of pain [interquartile range] between groups was 0 [0–2] versus 2 [1–3]; *P* < 0.001) [12] and primigravida mothers who had labor pain (mean ± SD of pain postintervention during active phase labor was 7.52 ± 0.23 versus 4.50 ± 0.23; *P* = 0.009) [13].

### 3.7. Clinical Effects of Herbal Compress in Inducing Lactation

Two studies evaluated the effects of herbal compress by measuring the duration from delivery to milk secretion in postcesarean mothers [20] and postpartum mothers [21] who had no milk production within 2 hours after delivery. The remaining study investigated herbal compress in mothers who had no milk production within 48 hours postpartum and used milk ejection score [22], as outcome measure, which was not comparable to the other two studies. Therefore, it was not included in the meta-analysis. The overall effect demonstrates that the application of herbal compress on breast in addition to a routine increasing milk production program has lessened the time from delivery to milk secretion compared with routine increasing milk production program (WMD −394.42 minutes; 95% CI −620.08, −168.77) with no evidence of heterogeneity amongst studies (*I* ^2^ = 0.0%, *P* = 0.38).

### 3.8. Adverse Effects of Herbal Compress

Safety outcomes were reported in only three studies [11, 12, 22] and all showed no adverse events associated with herbal compress.

## 4. Discussion

This systematic review and meta-analysis provided a comprehensive synopsis of the effects of herbal compress in various indications. Despite a lack of statistical significance, current evidence demonstrates a strong trend of better clinical benefits of Thai herbal compress compared with conventional therapies, such as NSAIDs, knee exercise, and hot compress in alleviating pain and improving difficulty to perform activities in patients with knee OA and muscle pain. However, Thai herbal compress was shown to be more effective than routine program in inducing milk production among postpartum mothers.

There are a number of similarities and differences of this systematic review and the previous Cochrane review. First, both reviews found limited number of studies to be included. Pooling results could not be determined in the Cochrane review [24] as the included seven studies comprised of seven different medicinal plants which were not comparable. The present review, on the other hand, has gathered evidences that focus on Thai herbal compress as the main intervention which were comparable as they reported similar outcomes for each indication used. In addition, at least two included studies used the same treatment/management as a comparator. These permitted a conduct of meta-analysis, which served as one of the strengths of this systematic review. However, a limitation of this systematic review lies within the diversity of methodological approaches observed across studies. They included the recipe of herbal compress, the procedure of intervention, and the number of sessions and duration of intervention. Moreover, quality of the majority of studies was relatively low as they were quasi-experimental studies which did not employ a randomization approach. In addition, due to the nature of intervention in applying herbal compress, it is not possible to undertake blinding method. Taking into account the relatively low quality of included studies, caution should be made when interpreting results derived from these studies.

Results from a recent systematic review [33] showed that heat and cryotherapy were recommended as nonpharmacological management of osteoarthritis in 10 out of 17 clinical practice guidelines. This suggested that heat from herbal compress may contribute to the clinical efficacy on the treatment of osteoarthritis. The effects of herbal compress in alleviating symptoms of knee osteoarthritis may also be associated with the main herbal ingredients which have been reported in several studies. For instance, results from a noninferiority trial in 367 patients showed that the extract of turmeric (*Curcuma domestica* or* C. longa*) 1,500 mg/day was as effective as ibuprofen 1,200 mg/day after a 4-week treatment of knee osteoarthritis [34]. Of note is that the effect found in this study was a result of orally administered curcumin, which is the main biologically active compound of turmeric, not a crude preparation as used in herbal compress. It is uncertain whether a topical application of turmeric herb would deliver clinical beneficial effects on knee osteoarthritis like oral curcumin extract due to the low solubility of natural curcumin that affect the absorption of this compound [35].* Zingiber montanum* is another potential ingredient in herbal compress that may play an important part in improving osteoarthritis symptoms. Results from a study on the combination of ginger (*Zingiber officinale*) and plai (*Zingiber montanum*) gel showed that this product was comparable to 1% diclofenac gel in relieving joint pain, improving problematic symptoms and quality of life in patient with osteoarthritis knees following a 6-week treatment [36]. This was consistent with the pooled effect presented in this review demonstrating that the reduction of pain in osteoarthritis patients treated with herbal compress was not significantly different from those receiving oral NSAIDs. However, anti-inflammatory effect of herbal compress used in the included studies could not be justified as it requires additional information on the measurement of anti-inflammatory related molecular/serum markers. A tendency of herbal compress to exhibit more pain reduction in patients with osteoarthritis compared with any other treatments/managements showed in this review warrant a conduct of further well design studies on a larger scale to confirm a clinical efficacy of this treatment.

Similar to the effects on osteoarthritis, heat was shown to be effective in treating patients with muscle pain [37]. However, evidence regarding the effects of main active herbal components on reducing muscle and labor pain was limited. Result from a related study on the effect of essential oil extracted from herbal compress active compound showed no additional benefit in reducing muscle pain compared with Thai massage [38]. These may in part explain the reason why the pool effect of herbal compress on muscle pain reduction in this review was not significantly different from hot compress.

In the present review, the pooled effect of herbal compress on labor pain reduction could not be drawn. However, findings from the individual studies indicated that herbal compress significantly reduced labor pain compared with usual care. Heat may be the main mechanism of action of herbal compress in improving labor pain as indicated in other studies [39, 40]. The pooled effect of herbal compress in increasing milk production found in this review was also consistent with findings from a previous study which showed that warming up the breast with a hot compress for 20 minutes significantly increased the amount of breast milk compared with nonwarmed breasts [41].

It appears that the effects of Thai herbal compress were mainly associated with heat and, in some extents, herb component. Effect of heat from applying herbal compress may explain why Thai herbal compress has exerted clinical benefits in two distinct indications, which were pain reduction and induce lactation. To identify additional effects from herbal component in both indications, a direct comparison between Thai herbal compress and hot compress is strongly advocated. However, it should be noted that the herbal compress recipes used among some of the included studies were slightly different and the quality of herbal compress varied as few were produced using good manufacturing practice (GMP) whilst some others were prepared in community with traditional method. A study on quality of herbal compresses produced in the community of eight provinces in the Northern of Thailand showed that the components of volatile oil in each product were different and contaminated with microbial organisms (7 out of 16, 43.8%) and cadmium (1 out of 16, 6.2%) [42].

Although no adverse events associated with herbal compress were reported in this review, it does not preclude the concern of safety issue over the use of this product. This is because there was a lack of attempt to investigate adverse effects of herbal compress among the majority of included studies as 10 out of 13 studies did not indicate that adverse events were systematically measured and reported. Findings from a report of adverse events of herbal compress revealed that 5 out of 600 patients were shown to have adverse reactions associated with herbal compress, that is, rash and urticaria [43]. Of these five patients, the adverse events reported in three were minimal and self-limited, whilst two patients reported moderate adverse reactions, and one patient required medical treatment. This suggested that the safety profile of Thai herbal compress should not be overlooked. To improve the lack of safety information from clinical studies, further studies on the effects of herbal compress should therefore include safety as one of the outcomes measured.

## 5. Conclusion

Current evidence suggested that Thai herbal compress might be more efficacious than standard/recommended therapy in patients with osteoarthritis and muscle pain. It could be considered as an alternative option for improving symptoms of these conditions especially when adverse effects from other treatments such as NSAIDs are an issue of concern. Thai herbal compress may also be used as a treatment of choice to induce lactation as the evidence suggested that herbal compress was more effective than usual care in promoting milk production in postpartum mothers who had no milk secretion. However, to consider Thai herbal compress to be incorporated into a practice guideline for each indication, we suggest that a consensus of standard recipe and practice need to be established and evaluated by further well-designed RCTs.

## Figures and Tables

**Table 1:** Characteristics of the included studies.

Study	Study type	Participants characteristics	Sample size	Male, %	Age, ^†^years	Herbal compress ingredients	Characteristics of herbal compress intervention			Control
							Detail of intervention	Number of sessions (duration)	Practitioner	
*Pain reduction *										
Artkarn, 2006 [10]	QE	Have recently been developed symptoms of back pain and knee pain with no prior history of using NSAIDs in the past 7 days	40	30.0	NR, range 25–55	The Institute of Thai Traditional Medicines Recipe^¶^	Steamed for 10 minutes then compressed after a 40-minute massage for 30 minutes on days 1, 3, and 5 with concomitant use of oral diclofenac 25 mg tid every day	5 (5 days)	A Thai traditional massage practitioner who has 5-year experience in using herbal compress	Oral diclofenac 25 mg tid every day
Chiranthanut et al., 2014 [11]	RCT	Have been diagnosed with knee OA according to the American College of Rheumatology for >3 months and had the VAS of pain in the range of 175–375 out of 500	60	27.0	NR, Int. 63.7 ± 6.1; Ctrl1 65.4 ± 9.8; Ctrl2 62.2 ± 9.5	The 225 g herbal balls contained dried herbs of *Zingiber montanum *rhizomes (40%), *Curcumalonga* L. rhizomes (10%), *Cymbopogon citratus* (DC.) Stapf leaves and leaf sheaths (10%), *Croton roxburghii* N. P. Balakr. leaves (10%), *Tamarindus indica* L. leaves (10%), *Citrus hystrix* DC. peels (5%), *Blumea balsamifera* (L.) DC. leaves (5%), *Vitex trifolia* L. leaves (5%), and camphor (5%)	Steamed for 20 minutes and compressed for 1 hour (changed the steamed herbal ball when the current one was slightly lukewarm) three times a week	9 (3 weeks)	A health professional practitioner who is trained in Suandok-style Thai massage	Ctrl1: massage for 1 hour three times a week; Ctrl2: oral ibuprofen 400 mg tid every day
Iampornchai et al., 2009 [12]	QE	Postpartum mothers who had back pain for both after labor and 24 hr after labor	100	0	NR, Int. 26.5 (23.0–33.0)^‡^; Ctrl 26.0 (21.7–30.0)^‡^	NR	Compressed after the court type Thai traditional massage for the total of 60 minutes	Once	Five trained Thai traditional medicine practitioners	Usual labor care including medication and exercise
Intarasukum and Kerdcharoen, 2011 [13]	QE	Primigravida mothers with singleton term who had labor pain	100	0	NR, Int. 22.6 ± 4.1; Ctrl 21.7 ± 4.8	*Zingiber montanum *500 g, *Citrus hystrix* DC. peels 100 g, *Cymbopogon citratus* Stafp 200 g, *Tamarindus indica* leaves 100 g, *Curcuma longa* L. 100 g, *Acacia concinna* (Willd.) DC. leaves 50 g, Salt 60 g, Camphor 30 g, and Borneo camphor 30 g	Start compressed on pelvis, coccyx, and waist when the cervix dilated for 4-5 cm, compress for 30 minutes and pause for 10–15 minutes then recompress for 30 minutes. Perform this cycle (compress and pause) until the cervix dilated for 8-9 cm, and then perform the final compress for 30 minutes	One course	The investigator (nurse)	Usual labor care
Lekutai et al., 2008 [14]	QE	Had been diagnosed with subacute knee OA with no plan for surgical treatment	89	NR	NR, range 51–55	Damnoen Saduak Hospital Recipe (*Zingiber montanum *500 g, *Curcuma longa* L.200 g, *Citrus hystrix* DC. peels 150 g, *Cymbopogon citratus* Stapf 100 g, *Tamarindus indica* leaves 100 g, Salt 50 g, Camphor 30 g, Borneo camphor 30 g)	Compressed for 30 minutes (changed the steamed herbal ball every 5 minutes) once a day	5 (5 days)	NR	Ctrl1: compressed with hot compress for 30 minutes once a day; Ctrl2: isometric exercise for 30 minutes once a day
Phromjuang, 2010 [15]	QE	Elderly (age 60–80 years) who had knee OA pain ≥6 months	40	NR	NR, range 60–80	*Zingiber montanum *500 g, *Curcuma longa* L. 200 g, Salt 1 tbsp, and Camphor 2 tbsp	Steamed for 10 minutes and then compressed for 30 minutes (changed the steamed herbal ball every 5 minutes) twice a week	8 (4 weeks)	NR	Usual care including oil massage and OA modern medicines
Puengsuwan et al., 2009 [16]	CT	Had been diagnosed with nonspecific low back pain	24	37.5	41 ± 8	Fresh herbs contain *Zingiber montanum*, *Curcuma longa* L., *Curcuma zedoaria* Roscoe, *Cryptolepis buchanani* Roem. & Schult, *Croton oblongifolius* Roxb. leaves, *Blumea balsamifera* (L.) DC. leaves, *Citrus hystrix* DC. peels, *Cymbopogon citratus* Stapf, *Tamarindus indica* leaves Salt, Camphor, and Borneo camphor	Steamed for 30 minutes then compressed for 20 minutes	Once	A Thai traditional massage practitioner	Compressed with hot compress for 20 minutes
Sathianrat et al., 2003 [17]	QE	Had myofascial pain syndrome with pain around neck, shoulder, and upper back for 2 days to 2 weeks	90	27.8	NR, range 20–69	The Institute of Thai Traditional Medicines Recipe^¶^	Compressed for 30 minutes on days 1, 3, and 5	3 (5 days)	A health professional practitioner who has 2-year experience in using herbal compress	Ctrl1: compressed with hot compress for 30 minutes on days 1, 3, and 5; Ctrl2: applied diclofenac gel at 7 am, 1 pm, and 9 pm everyday
Sukonthasarn, 2004 [18]	QE	Elderly (age >60 years) who had knee pain >1 year with no sign of inflammation at knee	75	NR	NR	NR	Compressed everyday	14 (2 weeks)	Village health volunteers who were trained in the use of herbal ball	Ctrl1: knee exercise 3 times a day; Ctrl2: knee exercise 3 times a day plus herbal compress once a day
Suwan, 2000 [19]	QE	Had been diagnosed with knee OA with no plan for surgical treatment	30	20.0	NR, range 40–79	*Zingiber montanum *500 g, *Curcuma longa* L. 500 g, Salt 5 tbsp, and Camphor 2 tbsp	Compressed everyday	7 (7 days)	The investigator (nurse)	Compressed with hot compress once a day

*Induce lactation *										
Listisit and Pakdeechot, 2009 [20]	QE	Postcesarean mothers who had no milk production within 2 hours postpartum	100	0	NR, Int. 32.0 ± 4.1; Ctrl 27.5 ± 5.6	NR	Compressed within 24 hours postcesarean	Once	The investigator (nurse)	Routine increasing milk production program (baby holding and breast feeding promotion every 2-3 hours)
Pakdeechot et al., 2010 [21]	QE	Postpartum mothers who had no milk production within 2 hours postpartum	100	0	NR, Int. 27.1 ± 5.6; Ctrl 26.8 ± 5.1	NR	Compressed within 24 hours postpartum	Once	NR	Routine increasing milk production program (baby holding and breast feeding promotion every 2-3 hours)
Trainapakul et al., 2010 [22]	QE	Mothers who had no milk production within 48 hours postpartum	46	0	NR, Int. 29.1 ± 6.4; Ctrl 27.7 ± 5.5	*Zingiber montanum *500 g, *Curcuma longa* L. 25 g, *Curcuma zedoaria* Roscoe 25 g, *Citrus hystrix* DC peels. 200 g, *Cymbopogon citratus* Stapf 100 g, *Tamarindus indica* leaves 50 g, Borneo camphor 5 g, Camphor 5 g	Steamed for 10 minutes and then compressed after a 10-minute breast massage for 20 minutes each breast	Once	A Thai traditional massage practitioner	Compressed after a 5-minute breast massage with mini hot bag for 5 minutes each breast

^†^Values presented as mean ± SD; ^‡^median (interquartile range); ^¶^ *Zingiber montanum *500 g, *Curcuma longa* L. 200 g, *Citrus hystrix* DC. peels 200 g, *Cymbopogon citratus* Stapf 100 g, *Tamarindus indica* leaves 300 g, *Acacia concinna* (Willd.) DC. leaves 100 g, Salt 1 tbsp, Camphor 2 tbsp, and Borneo camphor; CT, crossover trial; Ctrl, control; Int., intervention; NR, not reported; NSAIDs, nonsteroidal anti-inflammatory drugs; OA, osteoarthritis; QE, quasi-experimental; RCT, randomized controlled trial; tbsp, table spoon; tid, three times a day; VAS, visual analogue scale.

**Table 2:** Methodological quality assessment of the included studies.

Study	Risk of bias domain							Jadad score
	Sequence generation	Allocation concealment	Blinding	Incomplete outcome data	Selective outcome reporting	Other sources of bias	Overall risk of bias	
*Pain reduction *								
Artkarn, 2006 [10]	H	H	H	L	L	H	H	0
Chiranthanut et al., 2014 [11]	L	L	H	L	L	L	L	3
Iampornchai et al., 2009 [12]	H	H	H	L	H	L	H	0
Intarasukum and Kerdcharoen, 2011 [13]	L	H	H	L	L	H	H	2
Lekutai et al., 2008 [14]	H	H	H	L	L	H	H	1
Phromjuang, 2010 [15]	H	H	H	L	L	H	H	1
Puengsuwan et al., 2009 [16]	L	H	H	L	L	H	H	2
Sathianrat et al., 2003 [17]	H	H	H	L	L	H	H	0
Sukonthasarn, 2004 [18]	H	H	H	L	L	H	H	0
Suwan 2000 [19]	H	H	H	L	L	H	H	0
*Induce lactation *								
Listisit and Pakdeechot, 2009 [20]	H	H	H	L	L	H	H	0
Pakdeechot et al., 2010 [21]	H	H	H	L	L	H	H	0
Trainapakul et al., 2010 [22]	H	H	H	H	L	H	H	1

H, high risk of bias; L, low risk of bias.

**Table 3:** Outcomes of the included studies.

Study	Measuring tool	Herbal compress		Control		
		Before^†^	After^†^	Description	Before^†^	After^†^
*Knee OA— level of pain *						
Artkarn, 2006 [10]	Average VAS of pain when performing activities (score range 0–10)	5.5 ± 1.2	2.6 ± 1.2	Oral diclofenac	4.3 ± 2.0	2.8 ± 2.6
Chiranthanut et al., 2014 [11]	Total VAS of pain over the last two days when performing 5 daily activities (score range 0–500)	260.2 ± 68.9	61.6 ± 49.4	Oral ibuprofen	253.8 ± 63.4	69.2 ± 71.0
Lekutai et al., 2008 [14]	Average VAS of pain after wakeup, noon, 6 pm, and before going to bed (score range 0–10)	5.9 ± 2.0	2.8 ± 0.3	Hot compress Isometric knee exercise	5.4 ± 1.4 4.9 ± 1.7	2.5 ± 0.3 3.3 ± 0.3
Phromjuang, 2010 [15]	VAS of pain when performing activities (score range 0–10)	4.3 ± 1.8	1.7 ± 1.0	Usual care	3.9 ± 2.0	2.0 ± 1.1
Sukonthasarn, 2004 [18]	Average VAS of pain when performing 15 daily activities (0–10)	4.2 ± 1.0	2.8 ± 0.9	Knee exercise Knee exercise plus herbal compress	4.1 ± 1.3 4.4 ± 1.2	2.7 ± 1.2 3.0 ± 1.2
Suwan, 2000 [19]	Total VAS of pain when performing 13 daily activities (score range 0–130)	88.0 ± 24.0	36.6 ± 19.6	Hot compress	88.0 ± 26.2	53.2 ± 27.6

*Knee OA— difficulty in performing activities *						
Artkarn, 2006 [10]	Average VAS of difficulty in performing selected activities (score range, NR)		12.2 ± 16.5^‡^	Oral diclofenac		12.5 ± 14.7^‡^
Chiranthanut et al., 2014 [11]	Lequesne's functional index (score range 0–24)	13.2 ± 4.1	6.5 ± 3.7	Oral ibuprofen	12.7 ± 2.4	7.8 ± 3.3
Lekutai et al., 2008 [14]	Average VAS of difficulty in performing daily activities, during climbing up and down stairs, and during night time (score range 0–10)	6.0 ± 2.1	3.1 ± 2.6	Hot compress Isometric knee exercise	6.1 ± 1.4 5.1 ± 1.8	3.0 ± 1.9 2.4 ± 1.6
Sukonthasarn, 2004 [18]	Average VAS of difficulty in performing 20 daily activities	3.9 ± 2.7	3.2 ± 2.7	Knee exercise Knee exercise plus herbal compress	4.1 ± 2.5 4.0 ± 2.7	2.8 ± 2.4 2.5 ± 2.5
Suwan, 2000 [19]	Total VAS of difficulty in performing 22 daily activities (score range 0–220)	144.0 ± 35.9	76.2 ± 30.1	Hot compress	133.5 ± 30.3	86.4 ± 38.6

*Muscle pain—level of pain *						
Artkarn, 2006 [10]	Average VAS of pain when performing activities (score range 0–10)	6.1 ± 1.3	3.48 ± 1.1	Oral diclofenac	5.0 ± 1.9	2.2 ± 1.8
Puengsuwan et al., 2009 [16]	VAS of pain (score range 0–10)	3.4 ± 2.4	2.4 ± 2.4	Hot compress	3.7 ± 2.5	2.4 ± 2.1
Sathianrat et al., 2003 [17]	VAS of pain (score range 0–10)	5.7 ± 1.5	1.7 ± 1.3	Hot compress Topical diclofenac	5.0 ± 1.6 5.7 ± 1.7	2.1 ± 2.1 2.0 ± 2.1

*Labor pain—level of pain *						
Iampornchai et al., 2009 [12]	Pain numeric rating scale (score range 0–10)	5 (5-6)^¶^	4 (3–4.2)^¶^	Usual care	5 (5-6)^¶^	5 (3.8–5.2)^¶^
Intarasukum and Kerdcharoen, 2011 [13]	VAS of pain in early phase, that is, cervix dilated for 4-5 cm (score range 0–10)	6.2	4.5	Usual care	7.8	7.5
Intarasukum and Kerdcharoen, 2011 [13]	VAS of pain in late phase, that is, cervix dilated for 8-9 cm (score range 0–10)	6.2	6.4	Usual care	7.8	9.6

*Induce lactation *						
Listisit and Pakdeechot, 2009 [20]	Duration from delivery to milk secretion >1-2 drops, minutes		1,684.8 ± 540.7^‡^	Routine increasing milk production program		2,003.3 ± 575.3^‡^
Pakdeechot et al., 2010 [21]	Duration from delivery to milk secretion >1-2 drops, minutes		1,309.3 ± 535.3^‡^	Routine increasing milk production program		1,837.6 ± 901.1^‡^
Trainapakul et al., 2010 [22]	Milk ejection score (score range 0–4)	51.4^&^	54.6^&^	Breast massage followed by mini hot bag compress	42.1^&^	39.0^&^

^†^Values presented as mean ± SD; ^‡^mean difference; ^¶^median (interquartile range); ^&^mean rank; NR, not reported.

**Table 4:** The overall effects of herbal compress.

Comparator	Study	Mean difference [95% confidence interval]^†^	*P* value	Heterogeneity test			*I* ^2^, %
				χ^2^	df	*P* value	
*Knee OA—level of pain (various measures) *							
NSAIDs	Chiranthanut et al., 2014 [11]	0.222 [−0.400, 0.844]					
NSAIDs	Phromjuang, 2010 [15]	0.483 [−0.146, 1.112]					
**NSAIDs**	**Pooled effects (2 studies) **[11, 15]	**0.351 [−0.091, 0.793]** ^‡^	**0.120**	**0.33**	**1**	**0.563**	**0.0**
Knee exercise	Lekutai et al., 2008 [14]	1.430 [0.768–2.092]^¶^					
Knee exercise	Sukonthasarn, 2004 [18]	−0.100 [−0.720, 0.520]					
**Knee exercise**	**Pooled effects (2 studies) **[14, 18]	**0.660 [−0.839, 2.160]**	**0.388**	**10.92**	**1**	**0.001**	**90.8**
Hot compress	Lekutai et al., 2008 [14]	0.207 [−0.301, 0.714]					
Hot compress	Suwan, 2000 [19]	0.350 [−0.371, 1.072]					
**Hot compress**	**Pooled effects (2 studies )** [14, 19]	**0.254 [−0.161, 0.669]** ^‡^	**0.230**	**0.10**	**1**	**0.750**	**0.0**
**Combined comparators**	**Pooled effects (5 studies)** [11, 14, 15, 18, 19]	**0.419 [−0.004, 0.842]** ^‡^	**0.052**	**9.58**	**4**	**0.048**	**58.3**
*Knee OA—difficulty in performing activities (various measures) *							
NSAIDs	Chiranthanut et al., 2014 [11]	0.544 [−0.088, 1.176]					
Knee exercise	Lekutai et al., 2008 [14]	0.230 [−0.805, 1.265]^¶^					
Knee exercise	Sukonthasarn, 2004 [18]	−0.450 [−1.893, 0.993]					
**Knee exercise**	**Pooled effects (2 studies)** [14, 18]	**−0.001 [−0.842, 0.840]**	**0.998**	**0.56**	**1**	**0.453**	**0.0**
Hot compress	Lekutai, 2008 [14]	−0.059 [−0.565, 0.447]					
Hot compress	Suwan, 2000 [19]	0.610 [−0.123, 1.343]					
**Hot compress**	**Pooled effects (2 studies)** [14, 19]	**0.221 [−0.426, 0.868]** ^‡^	**0.504**	**2.17**	**1**	**0.141**	**53.9**
**Combined comparators**	**Pooled effects (4 studies)** [11, 14, 18, 19]	**0.224 [−0.128, 0.577]** ^‡^	**0.213**	**4.48**	**3**	**0.242**	**28.3**
*Muscle pain—level of pain (VAS of pain, score range 0–10) *							
NSAIDs	Sathianrat et al., 2003 [17]	0.300 [−0.539, 1.139]^¶^					
Hot compress	Puengsuwan et al., 2009 [16]	−0.280 [−1.613, 1.053]					
Hot compress	Sathianrat et al., 2003 [17]	1.130 [0.300, 1.960]					
**Hot compress**	**Pooled effects (2 studies)** [16, 17]	**0.525 [−0.842, 1.893]**	**0.452**	**3.10**	**1**	**0.078**	**67.7**
**Combined comparators**	**Pooled effects (2 studies)** [16, 17]	**0.135 [−0.574, 0.845]**	**0.708**	**0.52**	**1**	**0.470**	**0.0**
*Induce lactation (duration from delivery to milk secretion, minutes) *							
Routine program	Listisit and Pakdeechot, 2009 [20]	−318.500 [−501.015, −35.985]					
Routine program	Pakdeechot et al., 2010 [21]	−528.240 [−903.301, −153.179]					
**Routine program**	**Pooled effects (2 studies)** [20, 21]	**−394.425 [−620.084, −168.766]**	**0.001**	**0.77**	**1**	**0.381**	**0.0**

^†^Analyzed by weighted mean difference unless stated otherwise; ^‡^analyzed by standardized mean difference; ^¶^were selected for combined comparators analysis (for studies that had two comparator groups).

## References

[1] Jacobsen N., Salguero C. P. (2014). *Thai Herbal Medicine: Traditional Recipes for Health and Harmony*.

[2] The Institute of Thai Traditional Medicine (1995). *Thai Traditional Medicine Training Guide*.

[3] Chokevivat V., Chuthaputti A. The role of Thai traditional medicine in health promotion.

[4] The Institute of Thai Traditional Medicine (2009). *The Public Manual of Health Care with Thai Traditional Medicine*.

[5] Pitiporn S. (2012). *The Realm Memorandum IV: Herbs, Compres, Steam Soak, and Massage*.

[6] Aggarwal B. B., Prasad S., Reuter S. (2011). Identification of novel anti-inflammatory agents from ayurvedic medicine for prevention of chronic diseases: ‘reverse pharmacology’ and ‘bedside to bench’ approach. *Current Drug Targets*.

[7] Koontongkaew S., Meesuk L., Aupaphong V., Ayudhaya T. D. N., Poachanukoon O. (2013). Inhibitory effect of Zingiber cassumunar extracts on lipopolysaccharide-induced cyclooxygenase-2 and matrix metalloproteinase expression in human gingival fibroblasts. *Journal of Periodontal Research*.

[8] Chainani-Wu N. (2003). Safety and anti-inflammatory activity of curcumin: a component of tumeric (*Curcuma longa*). *Journal of Alternative and Complementary Medicine*.

[9] Lee H. J., Hyun E. A., Yoon W. J. (2006). In vitro anti-inflammatory and anti-oxidative effects of *Cinnamomum camphora* extracts. *Journal of Ethnopharmacology*.

[10] Artkarn C. (2006). *Effects of oral medication and oral medication combined with herb ball massage on pain in patients with musculoskeletal disorders [M.S. thesis]*.

[11] Chiranthanut N., Hanprasertpong N., Teekachunhatean S. (2014). Thai massage, and Thai herbal compress versus oral ibuprofen in symptomatic treatment of osteoarthritis of the knee: a randomized controlled trial. *BioMed Research International*.

[12] Iampornchai S., Poopong S., Nongbuadee S. (2009). Court-type traditional Thai massage and hot herbal compress: effectiveness in relieving early postpartum backache. *Journal of Thai Traditional & Alternative Medicine*.

[13] Intarasukum P., Kerdcharoen J. (2011). The effect of herbal ball hot compression on pain during labor among primigravida. *Journal of Health Science*.

[14] Lekutai S., Tansuwannon W., Sereechotchahiran S., Buakham P. (2008). Pain-relieving effects of hot herbal compress in patients with knee osteoarthritis. *Journal of Thai Traditional & Alternative Medicine*.

[15] Phromjuang K. (2010). The effects of Thai herbal compress on knee osteoarthritis pain among elderly. *Buddhachinaraj Medical Journal*.

[16] Puengsuwan P., Kamolrat T., Siritaratiwat W., Arayauuchanon P., Saetan O. (2009). A comparative study of heat effect between hot pack and Thai herbal ball on pain and physiological changes. *Journal of Medical Technology and Physical Therapy*.

[17] Sathianrat P., Rukkwamsuk P., Jantapink S., Tadphet S., Mala S., Buabubpa B. (2003). *Primary Efficacy of Applying Herbal Hot Comrpess to Muscle Pain Relief*.

[18] Sukonthasarn P. (2004). The effectiveness of hot herbal compression and knee exercise on knee joint pain reduction among the elderly. *Thai Health Promotion Journal*.

[19] Suwan P. (2000). *Effects of hot herbal compress on joint pain joint stiffness and physical disability among knee osteoarthritic patient [M.S. thesis]*.

[20] Listisit P., Pakdeechot S. (2009). The effect of increasing milk production program in mothers with cesarean section, Sakon Nakhon Hospital. *Journal of Sakon Nakhon Hospital*.

[21] Pakdeechot S., Morarad R., Sakontarat P. (2010). The effect of increasing milk production program on secretion time of colostrum in postpartum mothers, Sakon Nakhon Hospital. *Journal of Health Science*.

[22] Trainapakul C., Chaiyawattana M., Kanavitoon W. (2010). Effect of milk ejection performance of postpartum mothers after breasts massage and compression with mini hot bag and herbal compress. *Journal of Nursing and Education*.

[23] Department for Development of Thai Traditional and Alternative Medicine The 10th National Herb Exhibition. http://www.dtam.moph.go.th/index.php?option=com_content&view=article&id=344:pr0175&catid=8&Itemid=114.

[24] Cameron M., Chrubasik S. (2013). Topical herbal therapies for treating osteoarthritis. *Cochrane Database of Systematic Reviews*.

[25] Higgins J. P. T., Green S. (2011). *Cochrane Handbook for Systematic Reviews of Interventions Version 5.1.0*.

[26] Moher D., Liberati A., Tetzlaff J., Altman D. G. (2009). Preferred reporting items for systematic reviews and meta-analyses: the PRISMA statement. *Annals of Internal Medicine*.

[27] Gagnier J. J., Boon H., Rochon P., Moher D., Barnes J., Bombardier C. (2006). Reporting randomized, controlled trials of herbal interventions: an elaborated CONSORT statement. *Annals of Internal Medicine*.

[28] Jadad A. R., Moore R. A., Carroll D. (1996). Assessing the quality of reports of randomized clinical trials: is blinding necessary?. *Controlled Clinical Trials*.

[29] NICE (National Insitute for Health and Care Excellence) (2014). *Osteoarthritis: Care and Management in Adults*.

[30] NICE: National Insitute for Health and Care Excellence (2014). *Low Back Pain: Early Management of Persistent Non-Specific Low Back Pain*.

[31] Rosner B. (2010). *Fundamentals of Biostatistics*.

[32] DerSimonian R., Laird N. (1986). Meta-analysis in clinical trials. *Controlled Clinical Trials*.

[33] Brosseau L., Rahman P., Toupin-April K. (2014). A systematic critical appraisal for non-pharmacological management of osteoarthritis using the appraisal of guidelines research and evaluation II instrument. *PLoS ONE*.

[34] Kuptniratsaikul V., Dajpratham P., Taechaarpornkul W. (2014). Efficacy and safety of *Curcuma domestica* extracts compared with ibuprofen in patients with knee osteoarthritis: a multicenter study. *Clinical Interventions in Aging*.

[35] Henrotin Y., Priem F., Mobasheri A. (2013). Curcumin: a new paradigm and therapeutic opportunity for the treatment of osteoarthritis: curcumin for osteoarthritis management. *SpringerPlus*.

[36] Niempoog S., Siriarchavatana P., Kajsongkram T. (2012). The efficacy of Plygersic gel for use in the treatment of osteoarthritis of the knee. *Journal of the Medical Association of Thailand*.

[37] McIntosh G., Hall H. (2011). Low back pain (acute). *BMJ Clinical Evidence*.

[38] Ploylearmsang S., Arvipant R., Bhutakarm K., Suwanaphrom P. The sutdy of primary effectiveness of essential oil extracted from herbal compression on muscle pain relief.

[39] Fahami F., Behmanesh F., Valiani M., Ashouri E. (2011). Effect of heat therapy on pain severity in primigravida women. *Iranian Journal of Nursing and Midwifery Research*.

[40] Taavoni S., Abdolahian S., Haghani H. (2013). Effect of sacrum-perineum heat therapy on active phase labor pain and client satisfaction: a randomized, controlled trial study. *Pain Medicine*.

[41] Yiäÿit F., Çiäÿdem Z., Temizsoy E. (2012). Does warming the breasts affect the amount of breastmilk production?. *Breastfeeding Medicine*.

[42] Chonlasit K. (2008). *Standard and quality control of Lanna herbal hot compress [M.S. thesis]*.

[43] Nootim P. (2007). *Monitor of Adverse Reactions Associated with the Use of Herbal Compress*.

